# P-607. Immunogenicity and Tolerability of the Novavax Nanoparticle Influenza Vaccine (NIV) and COVID-Influenza Combination Vaccine (CIC)

**DOI:** 10.1093/ofid/ofae631.805

**Published:** 2025-01-29

**Authors:** Vivek Shinde, Susan Neal, Joyce S Plested, Tim Vincent, Mingzhu Zhu, Shane Cloney-Clark, Zhaohui Cai, Bridget Riviers, Farnaz Mahkhou, Anthony M Marchese, Iksung Cho, Louis F Fries, Wayne Woo

**Affiliations:** Novavax, Gaithersburg, Maryland; Novavax, Gaithersburg, Maryland; Novavax, Gaithersburg, Maryland; Novavax, Gaithersburg, Maryland; Novavax, Gaithersburg, Maryland; Novavax, Gaithersburg, Maryland; Novavax, Inc., Gaithersburg, Maryland; Novavax, Inc., Gaithersburg, Maryland; Novavax, Inc., Gaithersburg, Maryland; Novavax, Inc., Gaithersburg, Maryland; Novavax, Inc., Gaithersburg, Maryland; Novavax, Inc., Gaithersburg, Maryland; Novavax, Inc., Gaithersburg, Maryland

## Abstract

**Background:**

There is a public health need for annual immunizations against both SARS-CoV-2 and influenza viruses. We developed both a standalone saponin-adjuvanted (Matrix-M™) recombinant quadrivalent hemagglutinin (HA) nanoparticle influenza vaccine (qNIV), and a COVID and influenza combination (CIC) vaccine, comprising recombinant SARS-CoV-2 Spike (rS) (NVX-CoV2373), qNIV, and Matrix-M adjuvant.

**Table 1.**
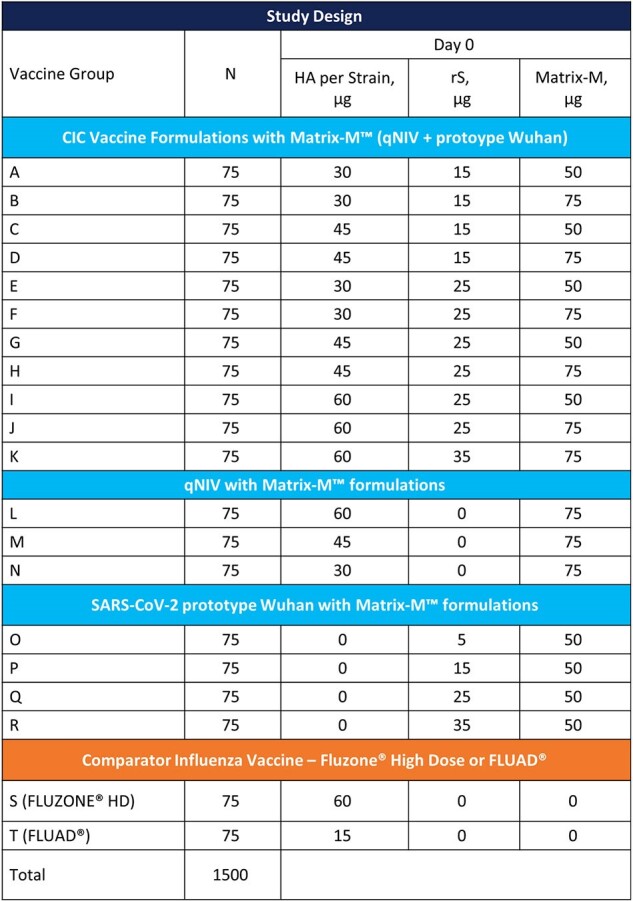
Study design

**Methods:**

Participants in Australia and New Zealand (1571 treated, including 864 in the CIC group) aged 50–80 years were randomized equally to receive one intramuscular dose of vaccine in 1 of 20 groups: either 1 of 11 different dose/formulations of CIC, 1 of 3 formulations of qNIV with Matrix-M, 1 of 4 formulations of standalone rS with Matrix-M, or 1 of 2 influenza vaccine comparators (Fluzone HD^®^ or FLUAD^®^) (**Table 1**). Immunogenicity assessments included anti-spike IgG, SARS-CoV-2 neutralizing antibody (vaccine-homologous and -heterologous strains), wild-type influenza HAI antibodies (vaccine-homologous strains). Reactogenicity was assessed for 7 days following vaccination and SAEs, AESIS, and MAAEs were assessed throughout the study.

**Table 2.**
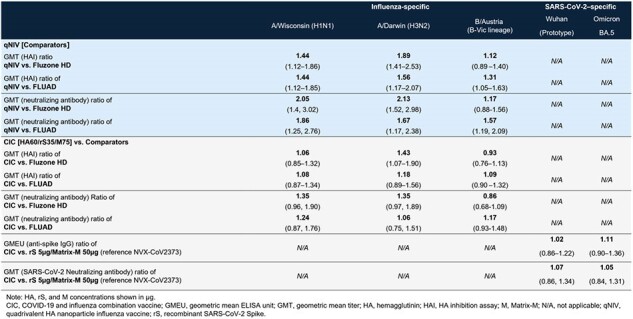
CIC and qNIV post-vaccination immunogenicity assessments (Day 21, per-protocol analysis set)

**Results:**

qNIV ([HA60/M75], n=75) HAI and neutralizing antibody responses were significantly higher than FLUAD and Fluzone HD against both vaccine-homologous A strains, particularly against H3N2 (**Table 2**). CIC ([HA60/rS35/M75], n=75) showed evidence of rS and HA antigen interference, but achieved anti-spike IgG and influenza HAI antibody responses that were comparable to both the standalone rS vaccine (NVX-CoV2373) and FLUAD /Fluzone HD, respectively (**Table 2**). All qNIV and CIC formulations evoked local and systemic solicited adverse events at rates and severities less than or comparable to FLUAD and Fluzone HD (**Figure 1**). There was no dose dependence of HA, rS antigens, or Matrix-M on tolerability. SAEs were infrequent in all groups.

**Figure 1.**
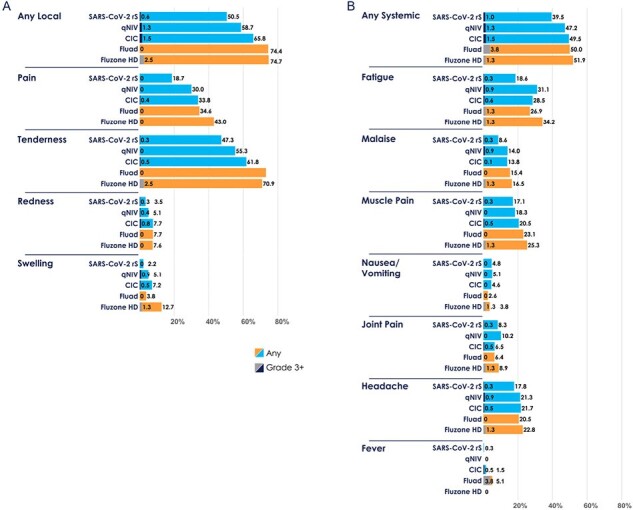
Solicited (A) local and (B) systemic TEAEs within 7 days of vaccination (safety analysis population)

**Conclusion:**

qNIV produced improved wild-type HAI antibody responses as compared to FLUAD and Fluzone HD against influenza A strains, notably against H3N2. CIC achieved both anti-spike IgG responses comparable to the authorized prototype NVX-CoV2373 rS vaccine and HAI responses comparable to licensed enhanced influenza comparators. CIC and qNIV had safety profiles comparable to Fluzone HD and FLUAD.

**Disclosures:**

**Vivek Shinde, MD, MPH**, Novavax, Inc.: employee|Novavax, Inc.: Stocks/Bonds (Public Company) **Joyce S. Plested, n/a**, Novavax, Inc.: employee|Novavax, Inc.: Stocks/Bonds (Public Company) **Tim Vincent, n/a**, Novavax, Inc.: Employee|Novavax, Inc.: Stocks/Bonds (Public Company) **Mingzhu Zhu, n/a**, Novavax, Inc.: employee|Novavax, Inc.: Stocks/Bonds (Public Company) **Shane Cloney-Clark, n/a**, Novavax, Inc.: employee|Novavax, Inc.: Stocks/Bonds (Public Company) **Zhaohui Cai, PhD**, Novavax, Inc.: employee|Novavax, Inc.: Stocks/Bonds (Public Company) **Bridget Riviers, Ph.D.**, Novavax, Inc.: Employee|Novavax, Inc.: Stocks/Bonds (Public Company) **Farnaz Mahkhou, Ph.D.**, Novavax, Inc.: employee|Novavax, Inc.: Stocks/Bonds (Public Company) **Anthony M. Marchese, PhD**, Novavax Inc: Employee|Novavax Inc: Stocks/Bonds (Public Company) **Iksung Cho, MS**, Novavax, Inc.: employee|Novavax, Inc.: Stocks/Bonds (Public Company) **Louis F. Fries, III, MD**, Novavax, Inc.: contractor for Novavax|Novavax, Inc.: Stocks/Bonds (Public Company) **Wayne Woo, MS**, Novavax, Inc.: employee|Novavax, Inc.: Stocks/Bonds (Public Company)

